# Factors promoting Iranian older adults’ spirituality: a qualitative content analysis

**DOI:** 10.1186/s12877-019-1146-7

**Published:** 2019-05-09

**Authors:** Fatemeh Hajinejad, Ebrahim Ebrahimi, Anneke de Jong, Maryam Ravanipour

**Affiliations:** 1 Department of Nursing, School of Nursing and Midwifery, Bushehr university of Medical Sciences, Rishehr Street, P.O.Box: 7518759577, Bushehr, Iran; 2 Heart Center Hospital, Bushehr University of Medical Sciences, Moallem Street, Bushehr, Iran; 3 Department of Nursing Studies, University of Applied Sciences, Heidelberglaan 7, post box 12011 – 3501AA, Utrecht, The Netherlands; 4 Department of Nursing, School of Nursing and Midwifery; And The Persian Gulf Tropical Medicine Research Center, The Persian Gulf Biomedical Sciences Research Institute, Bushehr university of Medical Sciences, Bushehr, Iran

**Keywords:** Spirituality, Older people, Qualitative content analysis, Iran

## Abstract

**Background:**

Spirituality plays an important role during old age, but reveals itself as a confounding mental health concept, which needs to be defined when providing spiritual care. The purpose of this study was to explore factors promoting Iranian older adults’ spirituality.

**Method:**

In a qualitative content analysis approach, we searched for the factors promoting spirituality among a selection of Iranian older adults. Totally, 22 people aged above 60 years old were interviewed using open-ended questions. The recorded interviews were then transcribed, and a coding process was applied based on a qualitative, conventional content analysis.

**Results:**

Three key factors were found to promote the spirituality among the older adults: 1) insight in personal belief, 2) spiritual socialization, and 3) peace of mind. Traditional dimensions, the cultural surroundings, and participants’ religious beliefs were apparent in each of the categories.

**Conclusion:**

Society’s role in making the seniors spiritual role models was very important; moreover the hereafter life on religious grounds seemed to be another important factor in reaching for high levels of spiritual and mystical perfection. Educational interventions based on the elders’ needs for spiritual empowering by health care professionals especially with regards to their spiritual based social interactions and reaching peace of mind, will comprise a major part of wellness approaches.

## Introduction

Life expectancy is increasing worldwide. Currently, persons older than 65 represent more than 12% of the population in the United States, and it is expected that this group will represent nearly 20% of the total U.S in 2030 [1]. In a 2010 census in Iran the number of aged people above 60 was estimated to be 8.2% of the whole population of the country [2].

There are essential differences between the Asian and Western world. Asians hold a more positive view of elderly people than the Western cultures do [3]. These views can influence the context of older adults and with that their perception of spirituality as well. There is a lesser degree of individualism present in Iranian families than for instance in Swedish ones. The elements of tradition and family tend to be dominant in Iranian families [4]. Cultural background in Iran promotes taking care of elderly people by younger members of the family. Therefore, it’s not well accepted in Iran to place old people in nursing homes [5, 6]. About 85% of Iranian elderly people live with their families and they consider their financial situation as average or poor [6]. The financial, health, welfare and emotional needs of Iranian elderly varies, as well as the fact that most of them are suffering from chronic diseases [7]. In Iran, the situation of female elderly concerning information, job opportunities, education, employment, social position and finances is worse compared with male elderly [6]. Female elderly are raised to be prepared for their traditional roles of being a mother and wife [8]. The major religion of most of Iranian’s elderly population is Islam and more than 99% of them are Muslim [6, 7]. Moreover, spiritual needs during old age plays an important role in empowering Iranian older people [9], and the element of spiritual power is more dominant in comparison with other elements during old age [10].

As individuals age, they seek meaning in their life. During elderly age, individuals have gained a high degree of spiritual awareness and recognition and it functions as a strong cultural force in elderly’s life [11]. In fact despite a decrease in physical strength in elderly people, the spiritual aspect becomes more intense and powerful [10]; and this can lead to personal growth and skills in individuals [12]. Spiritual closeness and connectedness was one of key experiences found to promote meaning and purpose in life among a selection of Norwegian nursing home residents [13]. On this basis, the studies revealed that spirituality has become a priority in societies [14].

The meaning of spirituality goes through changes with time and it is more of a structure related to physical and mental health [14]. Also as opposed to religion which has certain borders, unfortunately, spirituality remains a difficult concept to define and is focused more on connections than a specific definition [15]. An analysis of the definitions of spirituality in nursing research reveals inconsistencies and confounding mental health concepts. It is suggested to use a broader definition of spirituality and the definition preferably defined by patients [16].

Spirituality can be conceptualized as a characterological aspect of personality as findings indicated that spirituality is positively correlated with positive personality traits (i.e., extraversion) and negatively with negative personality traits (i.e., neuroticism) [17]. Spirituality exists in every individual and culture regardless of individuals’ affiliation to any specific religious group [18]. The assumption is that spirituality is a positive aspect of successful aging and is one of the predictors of an individual’s health during old age [19].

Several studies have verified the relationship between spirituality and positive health outcomes; spirituality provided participants with the resilience and strength to cope with living with a chronic illness [20]. In a qualitative study on female subjects with Acquired Immune Deficiency Syndrome (AIDS), the role of spirituality was stressed as a facilitator in self-care [21]. In Malaysia the elderly who were suffering from chronic diseases but held religious beliefs enjoyed a healthy state of mind in comparison with those who didn’t have such beliefs [22]. Spirituality, physical activities, and a healthy diet all contributed to a higher subjective physical well-being [23]. In order to provide older people with spiritual care, it is important to be totally aware of the evolution of faith and spirituality in elderly people. With some kind of spiritual help to ill elderly people, usually their threshold of tolerance for illness and problems will surpass their physical problems and they will find meaning in their lives [24].

Taking into consideration the relationship of the definition of spirituality and individuals’ cultural and traditional backgrounds and due to a lack of research on this subject in Iran, the aim of this study was to explore factors promoting Iranian older adults’ spirituality.

## Methods

This qualitative study was carried out in 2013 using the conventional content analysis approach. Content analysis as a research method is a normative reasonable method for describing a phenomenon and can help discover in-depth information on the participants and describe the quality of a phenomenon [25].

### Participants

The participants were 22 elderly individuals (14 women and 8 men) within a range of 60 to 82 years. The inclusion criteria were: be at least 60 years old, have the ability and tendency to express their experiences and feelings of spirituality and also were not suffering from any physical or mental problems. Exclusion criteria include not willing to participate in the study, or refusal to continue with the interviews. Participants were selected through purposeful and criterion-based sampling and it was continued till data saturation. Sample size in qualitative studies depends on required information [26] and is often low [27].

### Data collection

The elderly participants (informants) of this study were selected according to the mentioned inclusion and exclusion criteria. Initially, we selected some participants from ordinary community dwellers and through suggestions and referrals of either friends or relatives of the informants. All the informants were from city of Bushehr in the South of Iran. The interviews were carried out by a female researcher with 23 years of experience as an adult-gerontology nurse instructor. The interviewer had no care providing role in this study. The informants showed their consent for taking part in the study verbally and in written form. The first author of the study made phone calls with the potential participants to briefly describe the aim of the study. Once the participants gave their consents to take part in the study, appropriate time and place were determined by participants. On the day of the interview, the researcher visited the informants at their houses. Before starting the interview, the researcher, with respect to informants, established a proper communication with participants; thereafter the aims of the study, confidentiality of the information, and the recording of the interview were explained to them. The method of data collection in the study was the semi-structured personal interview. After obtaining written consent, the interview started with open questions which were determined in special panels such as: “How do you define spirituality? What are the criteria for spirituality? How would you like to be spiritual and why? What activities do you see as spiritual? What personal traits would you like to develop to be more spiritual?”, and based on their answers to these questions, more profound, complementary questions were asked to clarify the issue like “what do you mean by this? Can you explain more about this?” as probing and searching questions. The duration of the interview varied based on the participants’ tolerance and preferences from 30 to 90 min with a mean of 50 min. Since most of the participants were old and lacked the patience for long interviews, some of interviews were carried out in two separate sessions. In all, 28 interviews were carried out with 22 participants. All the interviews were recorded with an MP3 player.

### Data analysis

After conducting each interview, the text was transcribed verbatim and then coded [25]. Data analysis was performed by using the constant comparison technique and the Graneheim and Lundman’s qualitative content analysis approach. Accordingly, we took the following five steps for analyzing the data: 1. Transcribing the whole interview immediately after conducting it; 2. Reading the whole transcript for obtaining a general understanding about it; 3. Identifying meaning units and primary codes; 4. Categorizing similar codes into main categories; and 5. Identifying the main themes of the categories [28].

The primary analysis was performed by the first researcher. All of the interviews were recorded with a digital voice recorder and then were listened carefully, transcribed and typed word per word at the first occasion to keep relation with the data and the participants’ feelings. Afterward, the researcher reviewed the text and made notes of her first impressions. As this process continued, code labels emerged that were reflective of more than one key thought. These often came directly from the text and produced the initial coding scheme. For example a participant commented: “*I would like to reach spiritual heights but not materialistic ones. It’s very important and valuable to me when I can calm my friends and bring the light of faith back to them with a simple phone call*”; or another participant explained: “*I try to help the needy people, with buying ticket and participating in the charity concerts*” and the code “Performing spiritual activities based on social norms” emerged from these comments. Codes that were conceptually similar were placed in one cluster and the semantically related clusters were then organized into themes. For instance, the codes “Doing divine orders to achieve God’s satisfaction” and “Attempt to observe ethical principles” and “Performing spiritual activities based on social norms” were placed in a cluster as subtheme “Attempts to reach personal spiritual goals” and then this subtheme alongside with other subtheme were designated in the theme “insight in personal belief.” Two experts in qualitative analysis and subject matter (MR and AdJ) performed the transcript peer review and confirmed that 85% of codes and themes were accurate; in the case of discrepancies and different interpretations among researchers, several panels made up of the entire research team examined the coding process to agree upon a final version.

The interviews continued until saturation of the data. In this study, from the 19th interview on, no new code or data was obtained and the previous codes were repeated; to be on the safe side, 3 more interviews were done but no new information was obtained.

In order to increase the trustworthiness and rigor of the present study the following methods were utilized. Diversity of our participants will lead to an increase in confirmability of our data and for this purpose the participants were selected of both genders, of different life carriers and different levels of education. The first author had long experience of providing care to older peoples and hence, the criterion of prolonged engagement with the subject matter of the study was fulfilled. It means the pre-understanding of the older people’s situation in general by the interviewer, because of many years of clinical work with older patients made it easier to ask the appropriate follow-up questions. In addition time dedication for gathering the data, good interaction with the participants, returning the codes to the participants (member checks), verifying the authenticity of the codes interpreted from their interviews, checking and reviewing the data by colleagues as peer check were all used to increase the rigor of the data for the present study [25].

### Ethical considerations

The present study was approved, supported and financially sponsored by the Deputy of Research of Bushehr University of Medical Sciences (Grant No.22892) and Research Ethics Committee. The aim of the study was described to all participants. They were assured that their identity would remain confidential and that of the data in the interviews would be used solely for research purposes. They were also told that they would be allowed to quit the process any time without any repercussions. All the participants gave us their consent in both verbal and written form to take part in the study.

## Results

A total of 22 seniors (14 old women and 8 old men) participated in the study. The informants of the study were between 60 and 82 years old. All the informants were Muslims (Table 1).

**Table 1:** Demographic indicators of study informants

Informant #	Age	Education	Marital status	Perceived financial status	House ownership status
1	70–75	Illiterate	Widow	Average	Owner
2	60–65	Primary school	Married	Low	Rented
3	75–80	Illiterate	Widow	Average	Owner
4	70–75	Illiterate	Married	Average	Owner
5	65–70	Primary school	Widow	Average	Rented
6	70–75	Primary school	Widow	Average	Owner
7	70–75	Illiterate	Widow	Average	Living with other family members
8	70–75	Primary school	Married	Low	Rented
9	65–70	Primary school	Married	Good	Owner
10	80–85	Primary school	Widow	Low	Rented
11	70–75	Associate degree	Single	Good	Owner
12	65–70	Bachelor	Married	Good	Owner
13	60–65	High school	Divorced	Average	Owner
14	65–70	High school	Married	Average	Owner
15	60–65	High school	Single	Average	Owner
16	65–70	Bachelor	Married	Good	Owner
17	80–85	Illiterate	Married	Average	Living with other family members
18	65–70	Associate degree	Married	Average	Rented
19	70–75	Primary school	Widow	Low	Owner
20	75–80	Primary school	Married	Average	Owner
21	80–85	High school	Married	Average	Owner
22	80–85	High school	Married	Average	Owner

Coding and categorizing the interviews regarding the factors influencing spirituality pivoted around three main categories of ***insight in personal belief, spiritual socialization and peace of mind*** which each one of them had sub categories as shown in Table 2. Overall, spirituality according to Iranian older adults revealed as: because of their experience the informants gained insight into personal meaning of spirituality. Based on tradition, culture and religious norms the informants were spiritually socialized, gained insight in their personal beliefs of spirituality and connected this to reach peace of mind.

**Table 2:** Summary of the emerged subcategories and categories of concept of spirituality

Examples	Subcategories	Categories
• a sense of change in the direction of one’s needs in life towards spiritual examples,	Highlighting of personal spirituality	**Insight in personal belief**
• expansion of understanding and span of spirituality regarding God,		
• like to reach spiritual heights,	Attempts to reach spiritual goals	
• doing spiritual activities based on religious commands and traditions,		
• bring the light of faith back to the friends		
• developing kindness and forgiveness,	Getting socialized spirituality	**Spiritual socialization**
• observing moral principles in interaction with others,		
• keeping others’ secrets,		
• spiritual behavior in the family,	Fulfilling the role of a spiritual role model	
• benefiting the society and having an influence,		
• raise decent children who benefit the society		
• forgive and respond a bad deed with a good one,	Positive attitude towards life	**Peace of mind**
• ignore other peoples’ bad deeds,		
• leave those unsolved problems to “God”		
• a sense of freedom from worldly desires,	Guarantee of afterlife	
• tolerate the mortal nature of life,		
• expecting afterlife redemption and prosperity		

Bold entries are significant

### Insight in personal belief

Insight in personal belief has resulted from the process of personal spirituality cognition becoming more pronounced at old age, due to deliberating and finding spiritual examples (such as connecting to God and offering a huge amount of good and merciful deeds) based on their beliefs and in consequence attempt to reach that spiritual goals. The consciousness of the highlights of their spirituality was an internal understanding; and recognizing ways to increase spirituality acted as a driving force for moving to and enhancing insight. They believed that with time they had reached to this point of insight by contemplating about the different events of their own life and that this has led to a high capability in individuals to understand personal and social phenomena. They believed that this understanding is a personal feature that impacts the views, manners and choices of the individual. This category included the two subcategories of: the process of personal spirituality becoming more pronounced (named as highlighting of personal spirituality), and attempts to reach spiritual goals.

### Highlighting of personal spirituality

Personal spiritual insight during old age had sprung from a sense of self-awareness, an understanding of one’s spiritual needs becoming more accentuated, a sense of change in the direction of one’s needs in life towards spiritual examples and criteria; and the importance of reaching the apex of spirituality during old age. This personal spiritual insight can be considered as a source of energy or guidance in life for the individual to reach hope and peace of mind. The informants believed that while they have aged and have gone through different stages and changes in life, their understanding and span of spirituality regarding God and their relation with others has expanded. Through this they understand the true meaning of happiness. They did actually have faith in God but they feel it more at this age and they think that their faith will even increase as they get older. A 65 year old female participant describes her feelings as: “*At this age, I do whatever within my ability to help others, do good deeds and observe the principles of my religion. It’s important for me to do these as much as possible, because it brings good feelings to me. If it’s within my ability and I don’t do anything about it, I will have a troubled conscience and I will feel bad.*”

### Attempts to reach spiritual goals

From the elderly’s point of view, the attempt and search for spiritual goals is a type of practical insight on the path towards achieving high spiritual goals. As indicated by the participants’ views, spirituality is a path of life; In other words, it’s a lifestyle chosen by the individual through different ways such as spiritual investment or doing as much as good and merciful things and helps to others. In fact this insight had sprung from doing spiritual activities based on religious commands and principals, tradition and religious-based moralities of society and the feeling of joy from doing such activities. Attempting this path was the secret to the success and satisfaction of the elderly in the study. They desired to reach the most spiritual level but not materialistic ones. It’s very important and valuable to them when they can bring the light of faith back to the others with a simple act as an example of spirituality. A male 67-year-old participant added: “*Belief in God and trying to know him is a kind of spirituality. We have to do a lot of search to reach spirituality. This will lead to a positive attitude and view in the individual in all aspects of life whether scientific, practical or social. And this can be achieved through investment in life and the future of us and our children.*”

### Spiritual socialization

Spiritual socialization referred to the attention, observing and considering the spiritual norms of society which had been achieved through many years of experience in life and through respecting those norms. These experience in life comes from socializing agents (e.g., mothers, fathers, extended family members, peers and even their local society) in maintenance of spiritual values. This concept itself was made up of two other sub-categories of getting socialized spirituality and fulfilling the role of a spiritual role model.

### Getting socialized spirituality

Based on many years of experience and belief of the elderly participants in the effects of the nature of individuals on spiritual interaction with others, they had achieved a command of the spiritual norms of society. With the importance of such principles for them, in a way, their focus had shifted from themselves to the society and to other people, with an intention of helping them. Keeping people’s secrets, helping other people physically, financially and also having empathy for them and teaching children right from their childhood that they should not cause any harms to others and should do good deeds were some examples of getting socialized spirituality. Also the elderly used strategies such as loving other people, praying for other people’s health, developing kindness and forgiveness, observing moral principles in interaction with others and expecting themselves to be judged by the community, promoting social acceptance and friendship, and choosing the path of righteousness as socialized spirituality. A 65 years old female elderly says: “*I wouldn’t like to do bad things. Talking behind other people’s backs, pointless laughter and mocking others. I’d like to do things loved by God.*”

### Fulfilling the role of a spiritual role model

Fulfilling the role of a spiritual role model had been achieved through some kind of spiritual identity and feeling of empowerment by having reputation for spiritual behaviors such as tender heart, altruism, working conscientiousness, raising good children, benefiting the society and having an influence. Commonly spirituality to them means doing good things based on spiritual values to the others (e.g. altruism, hope, forgiveness, trust, love, the search for relational commitment, and promote social justice) and avoiding doing bad things (e.g. prevention of antisocial behaviors by emotional and behavioral self-regulation particularly in times of interpersonal conflicts). According to them when you do good deeds, help others, benefit your society and you are friendly and kind, you will be regarded as a role model by your family and others. From the participating elderly’s point of view, the spiritual position of the individual is actually his humane value which relies upon his purity of good intentions and good deeds. In fact, becoming a spiritual role model will occur after the individual’s deeds and spiritual behavior have become part of his identity and he is recognized by such manners. A 70 year old female elderly says: “*being honest and acceptance of the truth has increased my self-confidence and this has resulted from my spirituality and makes me feel strong. For this reason, nobody can talk behind other people’s backs when I’m around. Being virtuous, avoiding sins and respecting others’ dignity has made a name for me among friends and relatives. Even if I can’t help them financially, I will try to show my empathy for them and if I can I give them advice as well.*”

The elderly’s concerns for raising and having good children were obvious in their interviews. They believed that most of individuals’ behaviors and tendencies including helping others and caring about their duties come from the way they had been raised in the family. They believed that one of God’s blessings to humans is children and raising the children in the right manner will get you closer to God. In other words, children are God’s inheritance to us. The participants feel that they will be responsible for whatever they do even after their death. Raising them to be good children will guarantee a great position for them for now and in afterlife. If a family with such great attributes and sticking to moral principles and adhering God’s commands, raises good children, others will learn that if somebody is spiritual, their children will grow up to be good individuals as well. A 72 year old female elderly say: “*I do whatever it takes to raise my children and grand-children to be decent individuals. I do anything within my capability to achieve this and along the way I keep God in mind. Regardless of all the problems I have in my life, I have always asked God to bestow upon me patience and restraint and I thank God that I have managed to raise decent children who benefit the society and are not thieves, addicts or two faced… none of them. I always ask my children to be the best and of course it is the case with them because others have a positive view of them.*”

### Peace of mind

From participants’ point of view, having a positive attitude towards life against all the problems and misconducts in life, and guarantee their afterlife by behaving as spiritual, will achieve peace of mind.

#### Positive attitude towards life

As noted by the older people in this study, some kind of positive attitude towards life based on spiritual beliefs grows in the individual as a result of years of spiritual lifestyle. In fact, being spiritual means to be a good man, to forgive and to respond a bad deed with a good one which is better than revenge. Virtuous people who help others and bring them peace of mind will be responded accordingly by God and he secures their peace of mind and prosperity in return. This attitude will automatically guide the individual’s manners and choices based on spiritual beliefs; against others’ bad behavior and misconduct, they won’t refuse to forget them. In fact the older people’s attempts to achieve these goals lead to a growth in spiritual conscience and these results in peace of mind and inner calmness. A 73 year old female participant says: “*When I pay a visit to my neighbor who is ill, both my neighbor and I feel good about it. Doing good deeds brings me a sense of satisfaction and for this I thank God. Overall, paying a visit to relatives and friends is by itself spirituality and gets you close to God.*”

Regardless of trying to resolve their own problems, the majority of them also used to leave those problems to “God” and this had led them to feel more at ease and tolerate difficulties more easily. A 63-year-old female elderly states: “*If somebody had a spiritual view of life and keeps God in mind in every situation, she will not allow other people’s deviant views affect hers. Even if I find out that somebody talks behind my back, I’d leave them to God to put them back on the right path*”.

#### Guarantee of afterlife

Following the implementation and using the spiritual principles in one’s deeds, the elderly reach a clear conscience through dependence on God, a sense of freedom from worldly desires, belief in spirituality as a differentiating factor between humans and animals, following divine commands and observing spiritual norms of society. On the back of this clear conscience they achieve self-confidence, an ability to face difficulties and tolerate the mortal nature of life and they expected afterlife redemption and prosperity. Some strategies to reach the level of guarantee of afterlife was begin all the things by remembering God, when getting angry ask God for forgiveness, ask God to help them and bestow upon them patience. They also believed in afterlife in which they will be held accountable for their deeds in this world. A70 year old male participant points: “*Your fortune won’t last you forever. One has to rely upon God. As long as you are alive you have to pursue doing good deeds. You have to want to be alive in this world in order to have a good position in afterlife. You have to hold a positive view of other people. I feel free from burdens and sins when I forgive others for their misconducts and when I abstain from sinful activates.*” A 68 year old lady notes: “*Spirituality has led me to see beyond what life appears to be. I have become a virtuous person. I sense God within me and I feel the eternal joy and serenity*”.

## Discussion

Three main categories emerged from interviews with a selection of Iranian senior citizens participating in the study regarding the factors promoting spirituality: insight in personal belief through the promotion of highlighting of personal spirituality and attempt to reach spiritual goals; moreover, due to getting socialized spirituality and fulfilling the role of a spiritual role model they explained about second category named spiritual socialization. In the third category they explained that by positive attitude towards life and the guarantee of the afterlife they reach peace of mind. It also seems that spirituality in these senior citizens has a connection with the following of religious and moral codes in the society.

Regarding the emerged categories of this study and in comparison with several literatures, it found that most of spiritual criteria have attributed to things such as closeness to God, motivation, religious orientation, religious support and religious conflicts [29]. Nelson-Becker, Nakashima and Canda, have formulated eleven different aspects for spirituality including such areas as spiritual affiliation, spiritual behaviors, spiritual experiences, social support, and therapeutic change factors [30]. Female participants in a study revealed the concept of spirituality in five categories as follows: conflation, continuity, confidence, connection and caring [31]. Four measures of dispositional religiousness or spirituality were recognized as: general religiousness or spirituality, religious or spiritual commitment, religious or spiritual development, and religious or spiritual history [32]. Although in each of the above-mentioned studies a few aspects of spirituality have been investigated; in a larger scope, and based on our interpretation, it can be claimed that all these aspects can be analyzed in the same way. In other words, it seems that these studies center around three main domains: spiritual awareness (cognition), spiritual motivation (affection) and spiritual functions (behavior). Mattis, 2001 in examining the role of religion and spirituality in the relationships of African Americans, has presented a conceptual framework emerging out of a fundamental assumption that religion and spirituality are relational phenomena. This relational framework highlights the cognitive, affective, and behavioral correlates of religious and spiritual experience. They insist that religion and spirituality both stimulate and operate through a range of cognition, affect, and behaviors [33]. Comparing the findings of the present study with the findings of the study of Mattis, the cognitive domain was more noticeable in our study; on the other hand, emotional and behavioral areas were implicit in the findings of our study. Moreover, spirituality by some authors was defined as: it is the human experiences that seeks to transcend self and find meaning and purpose through connection with others, nature, and/or a Supreme Being, which may or may not involve religious structures or traditions. Reed theorized that human spirituality is a characteristic that is made visible by observable forms of connection within the individual to self (for example, personal integration), between individuals (friendship, trust), and of the individual with transcendent dimensions (mystical experiences) [34, 35]. In another study it was claimed that spirituality development can be gained through connection with self, others, world and reality [18]. This kind of categorization was somewhat more clear in our study findings, of course, with slightly different labels and focusing on God’s commands.

Regarding the insight in personal belief as our study finding and according to the informants’ experiences, spirituality comprises many perspectives at different levels of awareness. However, the results revealed that spirituality was viewed as inclusive and personal based on their insight in personal belief. Moreover, the development of spirituality and getting insight in their personal belief was a dynamic process in which a person became aware of meaning, values and purpose of their lives, including all relationships and attempt to reach personal spiritual goals.

From the developmental perspective, spiritual development can be understood as a universal human growth process that has multidimensional domains such as cognitive orientation, experiential and phenomenological dimension, existential well-being, and religiousness [36]. Alongside with the study informants’ beliefs regarding attempt to reach personal spiritual goals, ‘search’ has been described as an attempt to identify, articulate, maintain, or transform. It can be inferred that as an individual performs these steps, he begins to break his boundaries and grow and by doing that, he is actively and willingly creating a process of change and therefore involving his personal growth initiative [37]. Furthermore, the informants of our study pointed out to and attempted to follow personal, social, religious and moral codes which had originated from their understanding of spirituality. Spirituality generally is people’s demand for moral codes concerning the other individuals and groups’ deep experiences about the nature of ethics [18]. In this regard, tradition and culture, along with religious and spiritual beliefs, have even affected the sexuality desires of a selection of elderly women in Iran [38].

Based on the findings of our study in second category named spiritual socialization; in our study informants’ utterances, a shift from self to the others and the increasing importance of social interactions and the feedback of their spiritual behavior from society were seen. As they were seeking to boost spiritual behaviors and traits they were doing so to present a good spiritual model of themselves to the others and society that it was referred to their spiritual identity.

Regarding spiritual socialization, socializing agents (e.g., mothers, fathers, extended family members, peers) in the transmission and maintenance of religious and spiritual values within and across generations have an important role. Equally important, the mechanisms by which religious and spiritual socialization occur are concerned [33]. Families and communities are two key social systems for producing spiritual social capital for children and adolescents [36]. In a study carried out in Iran, culture and social environment were shown as an external effective factor of spiritual power. In the mentioned study leading others to senior citizens to provide them with spiritual guidance was introduced as an external sign of senior citizens’ spiritual power [10]. Attending religious ceremonies is the most important of any other social events that leads to an effective cognitive function [39]. In a study on Iranian seniors, it was revealed that seniors know that good rapport with each other is a source of social support and an important factor to improve physical, social and mental state [40].

Regarding the third emerged category in our study “peace of mind”, the findings uncovered that senior citizens due to spiritual ability and modeling in the face of several life problems, look on the bright side of issues and they made a reference to the peace of mind. In an investigation into some American senior citizens, the informants stated that they accepted and faced life difficulties and all adversity [41]. Asthmatic children and their families found psycho-intellectual management based on their spiritual beliefs as an effective method in reducing the negative burden of the disease on child’s mind, paying special spiritual and psychological attention to the child, maintaining the mental peace of family about child’s disease, and satisfaction about optimal treatment process [42]. In several studies, people have shown different views about arousing role of spirituality and religion in their health such as “I pray because I enjoy it”, “due to my kindness I seem nice in the eye of the public”, “I ask others to pray for me” [29]. Spirituality plays an important role in senior citizens’ adaptation to the old age and better quality of life [14]. In contrary to the Muslim informants of our study who believed in the hereafter, a life after death and reward due to good deeds done in this world is regarded as the best possible reward for a person, and it was inferred to as reaching a high level of self-actualization; it should be kept in mind that some other studies suggest that spirituality exists in all individuals with different degrees of religious and non-religious beliefs such theistic, atheistic, polytheistic, and other forms [18]. In fact in the current study, the concept of spirituality has its root in religious beliefs and implementation of religious practices in individuals (Islam is the case here). Literature says that people see spirituality in religion backgrounds and counts the closeness to God as the highest possible value for religious and spiritual thoughts that can bring about better health state [29]. Female informants in another study did not necessarily welcome death, but were accepting of their mortality and finitude with clarity due to strong connection with the Transcendence or God and they were grateful for all the good and bad happenings they had witnessed in their lives, their family and friends, and their longevity and survival [41].

## Conclusion

The point to be kept in mind about the current study is the importance of society’s role in making the seniors spiritual role models for others, family and society. The other point was that consideration to the hereafter life on religious grounds seemed to be another important factor in reaching for high levels of spiritual and mystical perfection and it is worth noting that not many studies have taken the point into account. Purposeful and criterion-based sampling in qualitative studies puts a limitation on generalization of the results and it just provide information about the investigated population. The other limitation of the study was carrying out into Muslim Iranian senior citizens and did not include other religions and senior citizens’ religious beliefs. Therefore, it is suggested further studies be carried out regarding senior citizens’ views on ethics and religion and exclusively investigated to arrive at better models to boost spirituality in golden years. As another recommendation, nurses and caregivers should facilitate their older clients’ search for peace of mind by learning skills that enable them to express personal beliefs as well as by supporting them to take part in relevant highlighting their personal spirituality and help that older adults to reach their spiritual goals. In fact, integrating an individual’s spiritual practice into their healthcare can help shape personalized medical care for older adults and improve health outcomes. Besides, based on the importance of society and religion in spirituality in Iranian older people, there is a need for much more research on developing tools and culturally appropriate interventions promoting spirituality with the focus on the insight in personal belief, and interpersonal and transpersonal connections especially in relation with religious belief in God. Educational interventions based on the elders’ needs for spiritual empowering by health care professionals especially with regards to their spiritual based social interactions and reaching peace of mind, will comprise a major part of wellness approaches.

## Abbreviations

AIDS: Acquired Immune Deficiency Syndrome

## References

[1] Eliopoulos C (2013). Gerontological Nursing.

[2] Motlagh M, Yazdani S, Taheri Tanjani P (2014). Elderly health profile in Islamic Republic of Iran. Tehran: Minisrty of health and medical education.

[3] Löckenhoff CE, De Fruyt F, Terracciano A, McCrae RR, De Bolle M, Costa PT, Aguilar-Vafaie ME, Ahn CK, Ahn HN, Alcalay L, Allik J (2009). Perceptions of aging across 26 cultures and their culture-level associates. Psychol Aging.

[4] Torres S (2001). Understandings of successful ageing in the context of migration: the case of Iranian immigrants in Sweden. Ageing Soc.

[5] Noroozian M (2012). The elderly population in Iran: an ever growing concern in the health system. Iran J Psychiatry Behav Sci.

[6] Tajvar M, Arab M, Montazeri A (2008). Determinants of health-related quality of life in elderly in Tehran, Iran. BMC Public Health.

[7] Teymoori F, Dadkhah A, Shirazikhah M (2006). Social welfare and health (mental, social, physical) status of aged people in Iran. Middle East Journal of Age and Ageing.

[8] McConatha JT, Stoller P, Oboudiat F (2001). Reflections of older Iranian women: adapting to life in the United States. J Aging Stud.

[9] Ravanipour M, Salehi S, Taleghani F, Abedi HA, Schuurmans MJ (2008). Power in Iranian elders: barriers and facilitators. Psychiatr Pol.

[10] Ravanipour M, Salehi S, Taleghani F, Abedi HA, Ishaghi SR, Schuurmans MJ, de Jong A (2013). Power resources of older people in Iran. Int J Older People Nursing.

[11] Stanhope M, Lancaster J. Foundations of nursing in the community: community-oriented practice. London: Elsevier Mosby; 2006.

[12] Birkenmaier J, Behrman G, Berg-Weger M (2005). Integrating curriculum and practice with students and their field supervisors: reflections on spirituality and the aging (rosa) model. Educ Gerontol.

[13] Drageset J, Haugan G, Tranvåg O (2017). Crucial aspects promoting meaning and purpose in life: perceptions of nursing home residents. BMC Geriatr.

[14] Lavretsky H (2010). Spirituality and aging. Aging Health.

[15] Edwards A, Pang N, Shiu V, Chan C (2010). The understanding of spirituality and the potential role of spiritual care in end-of-life and palliative care: a meta-study of qualitative research. Palliat Med.

[16] Reinert KG, Koenig HG (2013). Re-examining definitions of spirituality in nursing research. J Adv Nurs.

[17] Johnstone B, Yoon DP, Cohen D, Schopp LH, McCormack G, Campbell J, Smith M (2012). Relationships among spirituality, religious practices, personality factors, and health for five different faith traditions. J Relig Health.

[18] Nelson-Becker H, Canda ER (2008). Spirituality, religion, and aging research in social work: state of the art and future possibilities. J Relig Spiritual Aging.

[19] Cowlishaw S, Niele S, Teshuva K, Browning C, Kendig H (2013). Older adults' spirituality and life satisfaction: a longitudinal test of social support and sense of coherence as mediating mechanisms. Ageing Soc.

[20] Unantenne N, Warren N, Canaway R, Manderson L (2013). The strength to cope: spirituality and faith in chronic disease. J Relig Health.

[21] Tufts KA, Wessell J, Kearney T (2010). Self-care behaviors of African American women living with HIV: a qualitative perspective. J Assoc Nurses AIDS Care.

[22] Momtaz YA, Hamid TA, Yahaya N (2009). The role of religiosity on relationship between chronic health problems and psychological well-being among Malay Muslim older persons. Int Res J Med Sci.

[23] Boswell GH, Kahana E, Dilworth-Anderson P (2006). Spirituality and healthy lifestyle behaviors: stress counter-balancing effects on the well-being of older adults. J Relig Health.

[24] O'Brien ME. Spirituality in nursing standing on holy ground. London: Jones and Bartlett Publisher; 1999.

[25] Holloway I, Wheeler S. Qualitative research for nurses. Australia: Blackwell Science; 2002.

[26] Burns N, Grove SK (2005). The practice of nursing research, conduct, critique and utilization.

[27] Polit DF, Beck CT (2010). Essentials of nursing research, methods, appraisal, and utilization. 6th ed. United States: Lippincott Williams and Wilkins.

[28] Graneheim UH, Lundman B (2004). Qualitative content analysis in nursing research: concepts, procedures and measures to achieve trustworthiness. Nurse Educ Today.

[29] Hill Peter C., Pargament Kenneth I. (2003). Advances in the conceptualization and measurement of religion and spirituality: Implications for physical and mental health research. American Psychologist.

[30] Nelson-Becker H, Nakashima M, Canda E (2006). Spirituality in professional helping interventions. Handbook of social work in health and aging.

[31] Manning LK (2012). Articulating the ineffable: explorations into the spiritual lives of old women. J Relig Spiritual Aging.

[32] Moberg DO (2010). Spirituality research: measuring the immeasurable?. Perspectives on Science & Christian Faith.

[33] Mattis JS, Jagers RJ (2001). A relational framework for the study of religiosity and spirituality in the lives of African Americans. Journal of Community Psychology.

[34] Reed PG (1992). An emerging paradigm for the investigation of spirituality in nursing. Res Nurs Health.

[35] Buck HG (2006). Spirituality: concept analysis and model development. Holist Nurs Pract.

[36] Doe SS (2010). Children and adolescents in socio-cultural environments: towards a spiritual social capital theory. Currents.

[37] Ivtzan I, Chan CP, Gardner HE, Prashar K (2013). Linking religion and spirituality with psychological well-being: examining self-actualisation, meaning in life, and personal growth initiative. J Relig Health.

[38] Ravanipour M, Gharibi T, Gharibi T (2013). Elderly women’s views about sexual desire during old age: a qualitative study. Sex Disabil.

[39] Hill Terrence D. (2006). Religion, Spirituality, and Healthy Cognitive Aging. Southern Medical Journal.

[40] Bagheri-Nesami M, Shorofi SA (2014). Cultural and socio-economic factors on changes in aging among Iranian women. Global J Health Sci.

[41] Manning LK (2012). Spirituality as a lived experience: exploring the essence of spirituality for women in late life. Int J Aging Hum Dev.

[42] Renani HA, Hajinejad F, Idani E, Ravanipour M (2014). Children with asthma and their families’ viewpoints on spiritual and psychological resources in adaptation with the disease. J Relig Health.

